# Post-Fire Susceptibility to Brittle Fracture of Selected Steel Grades Used in Construction Industry—Assessment Based on the Instrumented Impact Test

**DOI:** 10.3390/ma14143922

**Published:** 2021-07-14

**Authors:** Mariusz Maslak, Michal Pazdanowski, Marek Stankiewicz, Paulina Zajdel

**Affiliations:** Faculty of Civil Engineering, Cracow University of Technology, 31-155 Cracow, Poland; michal@l5.pk.edu.pl (M.P.); goziolko@cyfronet.pl (M.S.); paulinazajdel22@gmail.com (P.Z.)

**Keywords:** fire, structural steel, steady-state heating regime, impact test, breaking energy

## Abstract

The change in the value of the breaking energy is discussed here for selected steel grades used in building structures after subjecting the samples made of them to episodes of heating in the steady-state heating regime and then cooling in simulated fire conditions. These changes were recorded based on the instrumented Charpy impact tests, in relation to the material initial state. The S355J2+N, 1H18N9T steels and also X2CrNiMoN22-5-3 duplex steel were selected for detailed analysis. The fire conditions were modelled experimentally by heating the samples and then keeping them for a specified time at a constant temperature of: 600 °C (first series) and 800 °C (second series), respectively. Two alternative cooling variants were investigated in the experiment: slow cooling of the samples in the furnace, simulating the natural fire progress, without any external extinguishing action and cooling in water mist simulating an extinguishing action by a fire brigade. The temperature of the tested samples was set at the level of −20 °C and alternatively at the level of +20 °C. The conducted analysis is aimed at assessing the risk of sudden, catastrophic fracture of load-bearing structure made of steel degraded as a result of a fire that occurred previously with different development scenarios.

## 1. Introduction

The suitability assessment of structural steel used in construction industry for possible further use after fire will be reliable only if, in the prepared expert opinion following the fire, the conventional inventory of permanent displacements and deformations found on site [1] is enriched with at least a set of appropriately interpreted results of experimental tests on basic material properties. In traditional approach those experiments are usually limited to analysis of changes in the yield limit and modulus of elasticity of the considered material, registered after cooling and related to the initial values of those parameters before the fire exposure. In the Authors’ opinion so narrowly focused research program seems to be insufficient, as it does not allow for real capability of formal verification, whether the steel after undergoing an episode of rapid heating due to the thermal action of fire followed by cooling, slow if occurring by natural convection in the furnace or rapid and sudden if induced by fire extinguishing action, did not become prone to brittle fracture. It is in such a situation in general a material structurally different than the same material described prior to the fire and this means that its strength properties may have undergone significant changes. The steel temperature during the fire episodes acting on it is often close to the temperature level programmed and applied during the manufacturing process to anneal and normalize its properties and thus may be capable of initiating various changes in its microstructure, in particular grain size growth and shape changes, chaotic grain reordering resulting in loss of advantageous banded structure developed during the manufacturing process [2], graphitization and spheroidization of grains [3] and most importantly creation of additional secondary precipitates [4]. Due to the random, and thus inconsistent with the scenario especially designed for the given steel grade in metallurgical processes, character of the thermal action induced by a fire, these changes are usually of detrimental character, weakening consistency of the structure [5] and in addition are permanent as being associated with the accumulation of gradually occurring local damage. Thus, the steel cooled after fire may, usually without exhibiting any changes visually observable during the traditional assessment conducted in order to evaluate its suitability for prolonged use, irrecoverably loose its plastic properties and thus turn into material prone to initiation and following unrestricted propagation of a brittle fracture. This type of threat is very dangerous to the potential user of a structure made of such steel, as it may result in sudden and unexpected failure, initiated without exhibiting any signs preceding the incident. Therefore, it is postulated to add the test of real susceptibility to brittle fracture of structural steel cooled after undergoing a fire incident, as a recommended test, to the set of basic tests conditioning rational conclusions regarding the possibility of its further application in building structures [6]. In Authors’ opinion the instrumented Charpy impact test [7,8,9,10,11] should be recommended in this field. The test of this type allows for determination and detailed analysis of the F−s curves depicting the relationship between the force breaking the tested sample and the displacement at the force application point. Identification on this curve of the range of changes revealing the risk of brittle fracture initiation, with an unstable and not effectively arrested propagation mode, may, in the Authors’ opinion, be treated as an unambiguous sign of an emerging treat. Such a statement in principle eliminates the tested steel from continued service. The proposed test seems to offer the best option for gaining knowledge in this domain by the expert performing the appraisal.

In the following part of this paper the way of interpreting the results obtained on the basis of this test is shown [12,13]. The detailed results of experimental research conducted by the Authors on selected steel grades commonly used in contemporary construction industry are also referred to [14]. The considerations presented here are aimed at demonstration and qualitative confirmation of compliance between the results indicated below, based on the analysis of F−s curves obtained in the course of tests with corresponding conclusions ensuing from the classical fractographic analysis of obtained fracture surfaces.

## 2. Interpreting of Impact Notch-Toughness Test Results in Terms of Assessing the Steel Susceptibility to Brittle Failure

Brittleness of metals and alloys is a consequence of:

- state of the metallic matrix—including crystal lattice of this matrix, as well as the shape and size of the constituent grains,
- secondary precipitates—including type, shape, size and quantity of these precipitates, their crystal lattices, locations—within the grains or at grain boundaries,
- energy state of the metallic matrix and intergranular boundaries—induced by plastic working or heat treatment of the alloy and its current temperature,
- presence of the internal stress concentrators.

Plasticity of metals and alloys is promoted by crystal lattices of matrix and secondary precipitates exhibiting multiple slip planes, regular equiaxed grains, high degree of intergranular boundary development (fine grains) and intergranular boundaries free of contaminants and gas microporosity. Brittleness of metals and alloys is promoted by crystal lattices exhibiting cleavage planes, coniferous grains and secondary precipitates, low degree of intergranular boundary development (coarse grains) and possible impurities at the grain boundaries.

Supersaturation of the metallic matrix with the alloy compound and ultra-dispersive intra grain secretions, usually created as the result of heat treatment intentionally designed for such alloys, crush induced by plastic treatment and even just lowering the alloy temperature (through the thus generated increase in the value of its characteristic yield point *R*_0.2_) decrease plasticity of the material, and thus increase its brittleness. Internal stress concentrators, impossible to eliminate in practice, additionally increase brittleness of the alloy. These concentrations may be due to the coniferous grain structure of metallic matrix or may be the result of secondary precipitates of various origins, cumulated in the microstructure of microcracks, rolled out impurities and gas pores, etc.

The steels exhibit metastable structure. Thus heating steel to the temperature exceeding the initiation temperature of diffusion processes usually results in changes in microstructure. These changes decrease the internal energy of the material, and thus lower the stresses in the crystal lattice. This usually occurs by the changes in grain shape and growth, separation of alloy compounds in the form of secondary phases (or by dissolution of secondary phases existing prior to heating), diffusive transport of impurities towards grain boundaries, annihilation of structural defects, combination and development of microcracks, intensive oxidation of material surface etc. The intensity of such processes depends on the amount of heat delivered to the material. They result in changes in mechanical properties of steel (relative to the initial state), usually of detrimental character. Deterioration of the strength properties as well as reduction of the plastic deformability are examples of such change. The chaotic course of heating and cooling incidents of steel exposed to real fire, difficult to reliably forecast and simulate in laboratory experiments, is undoubtedly a factor that further increases the unpredictability of the consequences of this type of exposure. Therefore, when considering such a scenario, one has to take into account the risk of the emergence of steel brittleness, which is very dangerous to the user, as this means the possibility of spontaneous cracking of the material in the presence of the external notches followed by catastrophic (not self-arresting) propagation of unstable cracks.

The F−s curve depicting the dependency between the breaking force *F* [kN] and displacement *s* [mm], registered at the force application point, is the basic result obtained for a steel sample subjected to an instrumented impact notch-toughness test. This curve is unequivocally related to relationship between the breaking work $KV=W_{t}\;\left[J\right]$ (usually interpreted as the energy dispersed during the breaking of the sample) and the same displacement. This energy is quantified by the area bounded by the F−s curve, thus the following holds:

(1) $$W_{t}=\int_{s=0}^{s_{i}}F\left(s\right)ds$$

**Figure 1:**
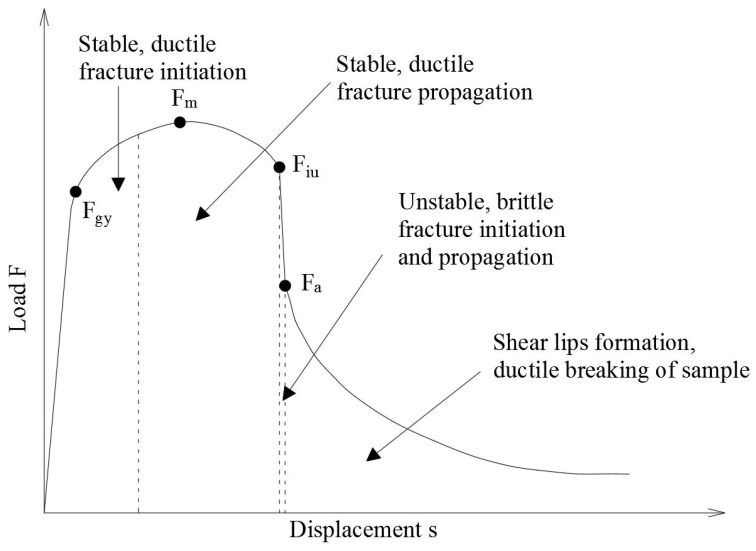
Theoretical F−s curve obtained during the impact notch-toughness test of steel specimens exhibiting mixed fracture.

Based on those curves conclusions are drawn regarding the susceptibility of a given material to the initiation of brittle fractures. The conditions for potential propagation of such fractures are also forecast, i.e., whether the propagation will be stable and to what degree will be susceptible to self-arrest [15]. The samples after the notch-toughness test may exhibit a fully plastic, completely brittle, or possibly in the most general case a mixed fracture [16]. Figure 1 depicts a typical theoretical F−s curve, usually obtained for a mixed fracture. The characteristic points indicating bounds for the subsequent phases of crack development are indicated there as well (ASTM E 2298-18 [17]).

The force $F_{gy}$ on this graph is usually associated with plastic initiation of developing fracture, while the force $F_{m}$ located at the apex of the F−s curve indicates, together with the accompanying displacement, the end of the stable fracture initiation phase. With further increasing displacement the already initiated fracture undergoes stable development phase, such that its propagation is accompanied by plastic deformation. The initiation of unstable fracture growth phase occurs only when the displacement associated with force $F_{iu}$ is reached. This phase is ended when the displacement associated with force $F_{a}$ is arrived at. At this moment the effective self-arresting of the fracture, so far progressing in an unstable manner, is initiated and as a consequence the specimen transits into the phase of plastic breaking [18].

**Figure 2:**
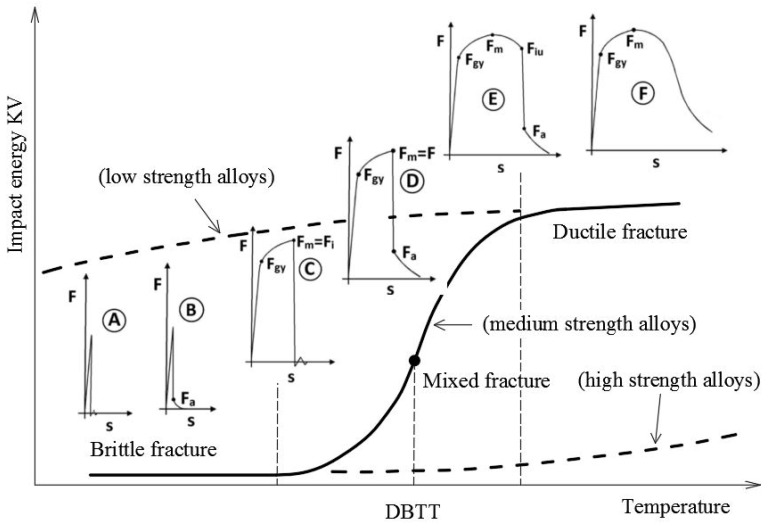
Location of standard F−s curves on KV−T graph (description in the text).

According to the EN ISO 14556 code [19] the shape of the F−s curve obtained during the experiment should be associated with one of the standard shapes, denoted by successive letters *A*, *B*, *C*, *D*, *E* and *F* (Figure 2). For the extreme shape, denoted by symbol *A*, only unstable, i.e., brittle fracture growth occurs during the whole process. The second extreme shape in this set is denoted by letter *F*. The process conforming to this shape does not exhibit unstable fracture growth at any moment. All the fracture development phases are stable then and this means that self-arresting of the fracture is fully effective. The larger the area under the F−s curve obtained during the test, however, determined for the displacement greater than the one associated with the limiting force $F_{a}$, the greater the capability of the sample material to efficiently arrest the unstable growth of previously initiated fracture. This in turn results in an appropriately larger area of ductile fracture observed on the fracture [20].

The code shapes of the F−s curves are assigned to particular locations, this time depicted on the graph exhibiting relationship between the specimen breaking energy (KV [J]) and the testing temperature (T [°C]). Inference on steel brittleness is here related to the limiting value of the breaking energy $KV_{min}=27\;\left[J\right]$ associated with ductile to brittle transition temperature (*DBTT*) [21]. The steels, for which in given testing temperature a breaking energy lower than $KV_{min}$ has been experimentally obtained (i.e., those conforming to the shapes *A*, *B*, *C* and *D*), in general exhibit dominating brittle character of fracture and as such do not exhibit the capability to effectively self-arrest initiated brittle fractures—therefore are not recommended for application in construction. The steels exhibiting such a capability (i.e., only those conforming to the shapes *E* and *F*) are characterized by the F−s curves located to the right of the threshold *DBTT* temperature [22].

## 3. Brittle Fracture Development Zones and Their Structural Conditioning

**Figure 3:**
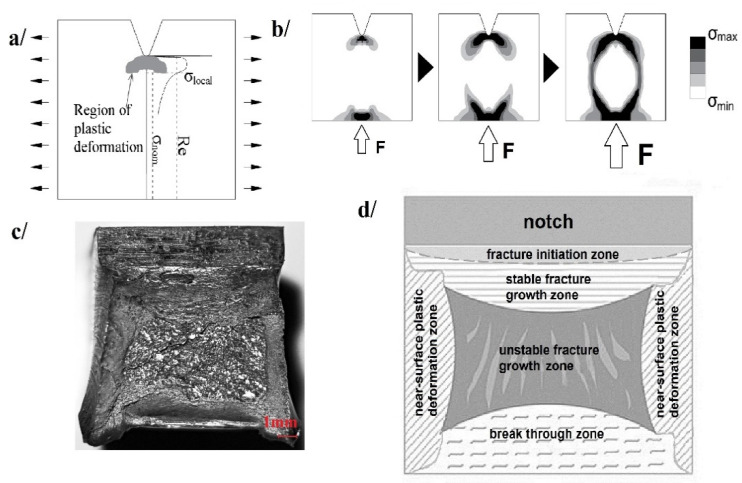
Notch effect on an ISO Charpy V steel sample: (**a**) local concentration of stresses under the notch crest as a result of pendulum strike, (**b**) numerical simulation of stress field development in a broken specimen, (**c**) typical fracture of mixed character, (**d**) identification of zones on the mixed fracture.

The typical fracture of a steel sample obtained during the impact notch-toughness test for a mixed fracture is depicted in Figure 3c. As shown, the fracture is initiated directly under the notch, though the pendulum strikes on the other side of the specimen (Figure 3b). This is due to the local concentration of stresses under the notch (Figure 3a). The following fracture development phases may be observed on the fracture surface. The surfaces of all the regions denoted in Figure 3d correspond in percent to the phases of this process, as registered on the F−s curve (Figure 1). As the number of degrees of freedom on the sides of the specimen is larger than on the inside, in these zones ductile fracture may occur. Unstable brittle fracture dominates the picture on the inside part of the specimen.

Brittle and plastic fractures should be associated with qualitatively different fracture mechanisms. On one hand inclusions such as for instance carbides, oxides, borides, nitrides or silicates are incoherent with the crystallographic matrix and thus constitute a crystalline void. Their presence induces the fracture mechanism dominated by brittle fracture. On the other hand, presence of manganese sulfites in the steel structure may lead to domination of a plastic fracture. The difference in action of both inclusion groups indicated above is that the thermal expansion coefficients of inclusions belonging to the first group are lower than the analogous coefficients of the crystallographic matrix, thus the crystallographic matrix “tightens” over an inclusion in a sense. In the case of manganese sulfites the value of corresponding thermal expansion coefficient is higher than the value of analogous coefficients for crystallographic matrix, thus the inclusion tightens itself within the matrix and a „void” is developed. The oxides and silicates (and the remaining inclusions belonging to this group) represent good brittle fracture initiators, as continuity between dislocation network in the crystallographic matrix and brittle particle is assured, while manganese sulfites in such approach represent initiators of voids leading to initiation of ductile fracture [23,24]. Brittle fracture observed on test samples cooled after prior exposure to fire temperature may also be explained as a consequence of unwanted so-called sigma phase separation in their structure (this pertains only to certain acid resistant steels of grade 18/10). In the theoretical description, the brittle fracture process in carbon and low alloy steels usually comprises three stages, in the following sequence: crack nucleation on particles (of for instance carbides), propagation of these cracks on phase boundaries between carbides and ferrite and finally propagation of the fracture on the first ferrite-ferrite grain boundary [25].

## 4. Description of the Impact Notch-Toughness Test Conducted

The purpose of the experimental research conducted by the Authors may be stated as verification of the suitability for use of selected steel grades commonly used in construction industry after undergoing a fire event. The Authors intend to verify, whether the samples made of these steel grades, cooled after preceding exposure to fire temperature, will prove prone to brittle fracture, as this would disqualify these steels from potential further service, especially under load. The following steel grades have been selected for the detailed investigation:

- S355J2+N steel—low alloy two phase ferritic-pearlitic with stranded perlite, raised manganese content, subjected to normalization, representing a group of steels exhibiting low sensitivity to short time thermal action (in the temperature below 350 °C),
- 1H18N9T steel—Cr-Ni high alloy, single phase, acid resistant, austenitic with carbon stabilized by the addition of titanium, supersaturated with a temperature of approximately 1100 °C, representing a group of steels recommended for use at the temperature values not exceeding 600 °C (due to the risk of unwanted sigma phase separation at the temperature range of 650 °C—850 °C),
- X2CrNiMoN22-5-3 steel—high alloy, two phase, austenitic-pearlitic, of the duplex type, supersaturated with a temperature of approximately 1050 °C in water, representing a group of duplex steels recommended for use at the temperature values not exceeding 300 °C (due to the occurrence of unfavourable 475 °C brittle failure phenomenon).

As indicated above, these steels are considered to be representative for the whole groups of steel grades applied in construction with respect to experimental results expected.

**Figure 4:**
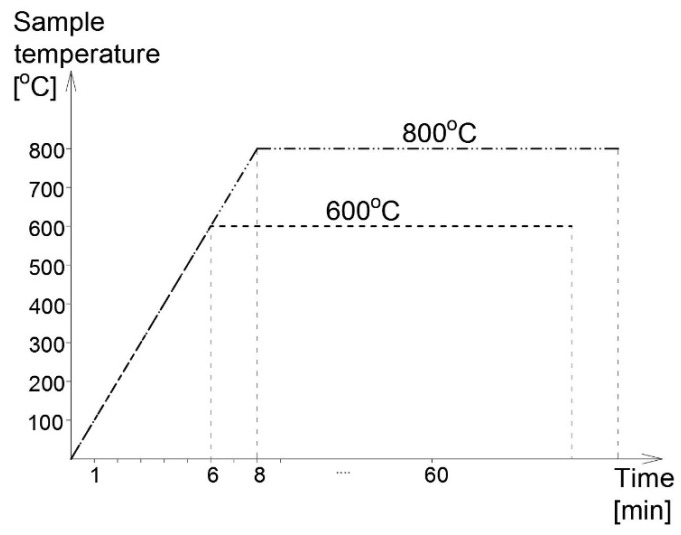
Fire action simulated during research.

In our research the susceptibility of steel to brittle fracture after surviving a fire incident has been verified on an instrumented Charpy impact notch-toughness test [26], with the physical action of fully developed fire was simulated by a proper preparation of test samples [14]. Prior to the test, these samples have been subjected to thermal action of formalized course. During the first phase of this process these samples had been heated to the temperature of 600 °C (first set) or 800 °C (second set), respectively, then kept for 60 min at this constant raised temperature, conforming to the conventional steady state heating regime (Figure 4). Following the conclusion of the heating the samples have been cooled to the room temperature. During the tests, for comparative purposes, two alternative cooling scenarios have been applied, namely slow cooling of samples in the furnace, simulating self-extinguishing of a fire and cooling in the water mist corresponding to the intervention of a fire brigade.

**Figure 5:**
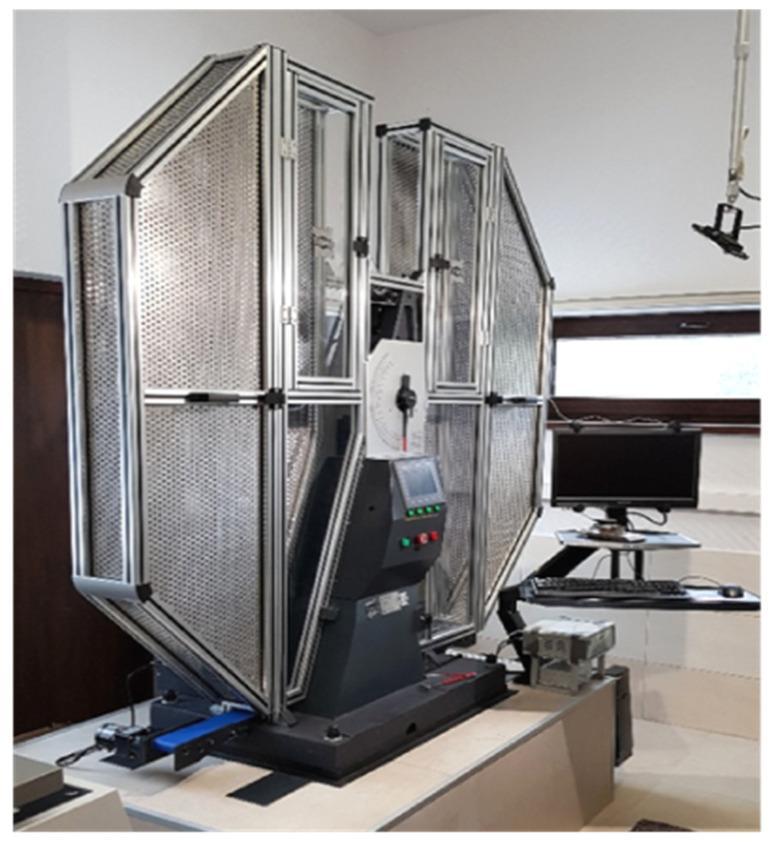
Instrumented Charpy pendulum used to conduct the tests.

The impact notch-toughness tests of samples prepared in advance have been conducted conforming to the recommendations of the EN ISO 14556 code [19] on an instrumented Charpy pendulum JB-W450E-L (Figure 5), having the potential energy of 450 J. The R8 (American type) pendulum hammer has been selected for the tests, as it is understood that in construction industry an impact by a flat surfaced object is more probable (with respect to the classical R2 pendulum hammer of European type) [27]. The hammer was equipped with a transducer used to measure the force hitting the specimen and the displacement of the force application point accompanying the load application sequence was measured with an encoder. The signals delivered by both transducers were collected and processed by a data logger of high sampling frequency and finally analyzed by specialized software designed for that purpose. The results of each test were recorded and depicted on automatically generated graphs displaying the force, energy and displacement as functions of time or force and energy as functions of displacement. The software automatically indicated on those graphs the locations of characteristic limit points, as well.

**Figure 6:**
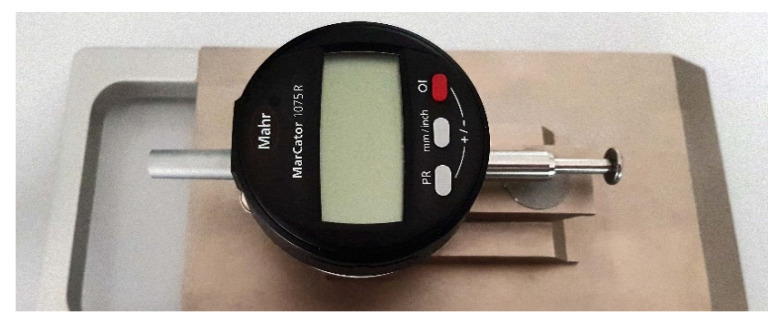
Measurement device used to measure the longitudinal expansion *LE*.

The destructive tests have been conducted at the temperature of +20 °C (simulating summer conditions) and alternatively at the temperature of −20 °C (to simulate winter conditions). Each time the F−s curve (Figure 1) and the corresponding $W_{t}-s$ graph have been recorded. In addition, the longitudinal expansion *LE* [mm] of broken samples has been measured, as well (Figure 6).

Performed tests included 30 qualitatively different measurement cases. For each of the considered steel grades and each of the two temperature values four independent test cases related to steel after initial heat treatment (two heating regimens times two cooling scenarios) and one case related to the untreated steel in the virgin state (without initial heat treatment) have been conducted. Each measurement case consisted of test performed on six independent steel samples, to assure sufficient reliability of the final estimate obtained. Final test results for a measurement case have been averaged over these six samples. Thus, overall 30 × 6 = 180 independent impact notch-toughness tests have been conducted.

Reduction of the data sets obtained during the experiment to F−s and $W_{t}-s$ graphs averaged for each measurement case and averaged value of the longitudinal expansion *LE* allowed for simplified treatment in the analysis of the random variability in the impact toughness sought. The final results have been archived, using three digit notation scheme applied to separate test cases, with consecutive digits interpreted as shown in Table 1. The case denoted with single digit 1, 2 or 3 in this notation denotes the set consisting of six individual tests performed on steel remaining in the initial virgin state (a sample which did not undergo the preliminary heat treatment).

All the considered tests related to the S355J2+N and 1H18N9T steels have been conducted on full size ISO Charpy V-10 samples. In the analogous cases, related to X2CrNiMoN22-5-3 duplex steel, the samples had to be reduced in size to ISO Charpy V-7.5 [28], as the potential energy of the 450 J pendulum proved to be insufficient to break these samples both in the initial state, as well as after the heating simulation conducted in the temperature of 600 °C during one hour and rapid cooling in water mist.

The impact notch-toughness test samples made of the steel grades analyzed are coded in a coding system shown in the Table 1.

**Figure 7:**
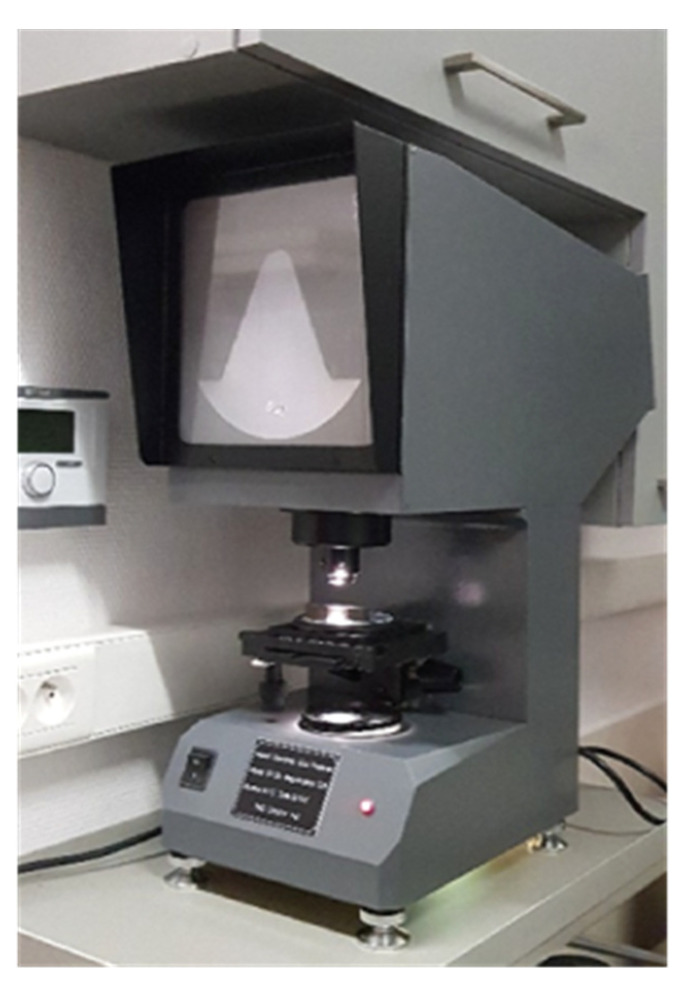
The notch profile projector XT-50 used during the tests to verify whether the notch size tolerance criteria have been observed.

The breaking energy in the notch-toughness test is a local property concerning the cross section directly under notch. The notches in the analysed samples have been made prior to the thermal treatment simulating fire action. This procedure had to be observed, as should these notches be made after heat treatment the structural changes generated by the temperature in the cross section authoritative for the crack initiation would be largely removed. A new factory made Charpy V cutter has been used to make the notches, and the profile obtained remained within the limits prescribed by the codes. Observation of the notch profile on the samples in the initial state, on the notch profile projector XT-50 (Figure 7), has shown, that the notches made were located just below the upper tolerance limit prescribed by the code EN-ISO 148-1 [29]. Repeated observation of the notches after heat treatment and removal of the scale induced by steel surface oxidation revealed increase in their size, so in a few cases their size slightly exceeded the tolerance allowed. The change in the notch profile and dimensions related to the formation of surface scale is a natural result of sample oxidation due to fire. The amount by which the upper tolerance limit has been exceeded, observed in the experiments presented here, was very slight in the Authors’ opinion and did not affect the final evaluation of the breaking energy measured in the test (it is equivalent to approximately 1.4 times the friction losses of the pendulum having an initial energy of 450 J on its bearings and in the air). The possible additional corrections of the notch profile performed after heat treatment would result in falsification of the structural changes induced by this treatment, and this most probably would distort the final results of this study. Let us note as well, that according to the provisions of the ASTM E23-92 standard [30], changes of this type may result in an increase in the breaking energy measured in the test only by about 2–3 J in brittle samples and by a negligible value in ductile samples. Therefore, it seems that the value of the potential measurement error identified in the presented study does not have a significant impact on the final conclusion.

## 5. Detailed Analysis of the Obtained Results

### 5.1. S355J2 + N Steel

**Figure 8:**
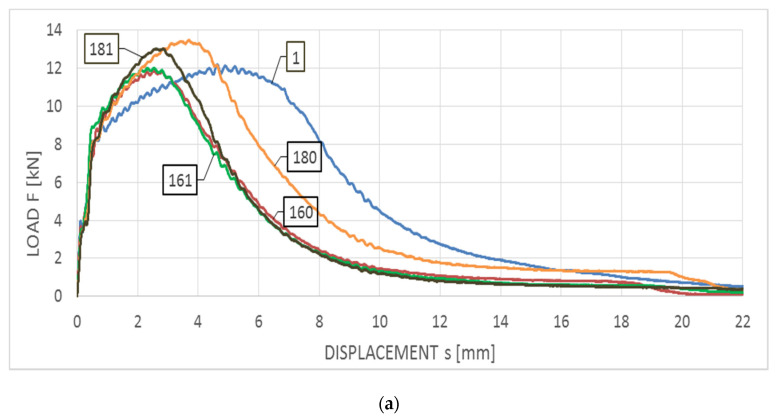

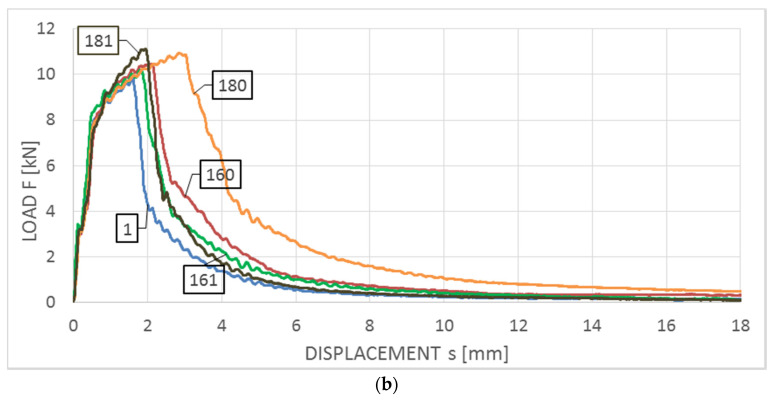
Averaged F−s curves obtained on samples made of S355J2+N steel: (**a**) during tests conducted in +20 °C, (**b**) during tests conducted in −20 °C.

The averaged F−s curves obtained for this steel at the temperature of +20 °C are depicted in Figure 8a, while those obtained at the temperature of −20 °C are depicted in Figure 8b, correspondingly. The first group of these results (depicted in Figure 8a), after comparison against sample curves included in the code (according to EN ISO 14556 [19]), may be qualified as conforming to the F type curve, denoting plastic zone devoid of unstable crack growth area. The second group (depicted in Figure 8b) corresponds to the D type curve, unequivocally revealing the area of unstable crack growth. Let us note, however, that in this case the unstable crack growth area had only limited reach, as the material exhibited the capability of effectively arresting the brittle crack growth by the plastic self-restraint.

**Figure 9:**
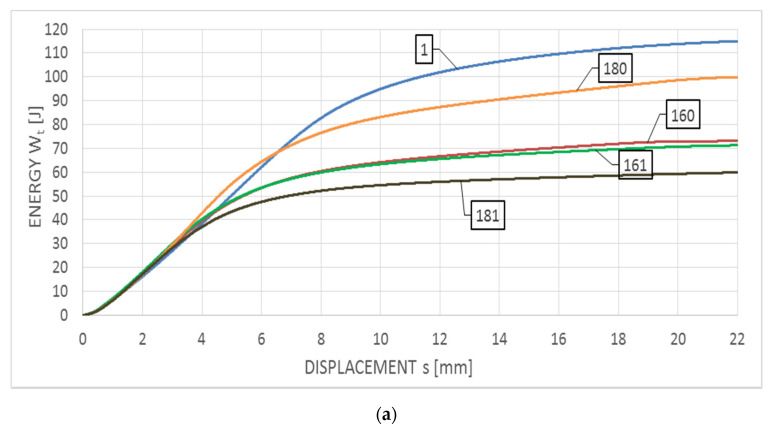

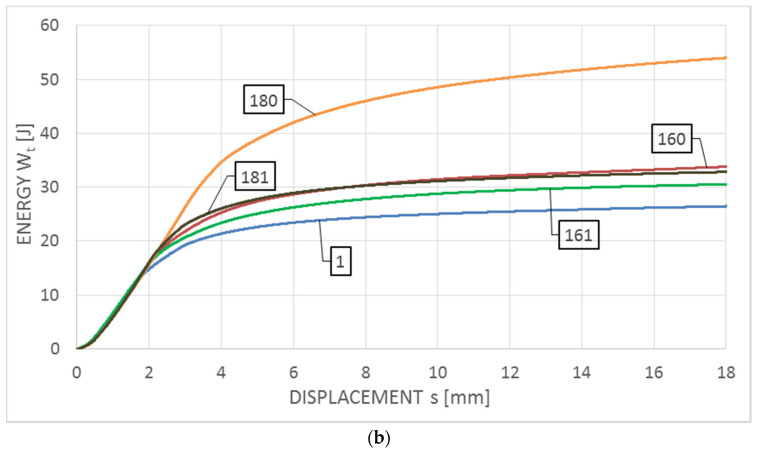
Averaged $W_{t}-s$ curves obtained on samples made of S355J2+N steel: (**a**) during tests conducted in +20 °C, (**b**) during tests conducted in −20 °C.

Figure 9 depicts $W_{t}-s$ relationships obtained for S355J2+N steel and corresponding to the F−s graphs shown in Figure 8 (curves depicted in Figure 9a correspond to graphs presented in Figure 8a, while analogous curves of Figure 9b correspond to graphs presented in Figure 8b). It has to be emphasized, that the considered steel in the initial state and in the testing temperature of +20 °C (Figure 9a—group of samples denoted with digit 1) exhibited higher values of the breaking energy $W_{t}$ (117 J) as well as averaged longitudinal expansion LE (1.94 mm), when compared to the remaining samples made of the same steel, but subjected to the preliminary heat treatment. However, in the case of tests conducted at the temperature of −20 °C the opposite proved to be true. Both the averaged breaking energy $W_{t}$ (26 J) and the averaged longitudinal expansion LE (0.56 mm) determined on samples in the initial state (Figure 9b—a group of samples denoted with digit 1) were lower than the corresponding values determined on samples subjected to the preliminary heat treatment.

In addition, the cooling mode significantly affected the behavior and properties of the samples analyzed, if these samples have been subjected to heating only at the temperature of 800 °C (with respect to the impact notch-toughness test conducted at both temperatures of +20 °C and −20 °C). Heating to so high temperature usually results in partial austenitization of the steel structure [31]. The following slow cooling of the sample on the open air (sample marked as 180) leads under those circumstances to development of structure containing spheroidal carbides uniformly distributed in the ferritic lattice, and this results in increased plasticity and impact strength. Fast cooling with water (sample 181) has its consequences in carbon oversaturation of a part of ferrite formed from austenite and hardening of steel, and thus decreased plasticity and impact strength. This phenomenon has not been observed on samples heated to 600 °C only. The shapes of curves associated with samples 160 and 161 were very similar, as can be observed on both Figure 9a,b. For the reasons listed above the results obtained for the specimens denoted with 180, regardless of the temperature at which the impact test has been made, definitely stood out from all the other simulations conducted for this steel. The remaining scenarios, both those associated with the heating temperature of 600 °C (regardless of the cooling mode) and the heating temperature of 800 °C followed by cooling with water yielded similar results expressed by similar shape of the F−s curves and thus estimated values of the breaking work $W_{t}$ (60–74 J for the tests conducted at +20 °C and 31–35 J for the tests conducted at −20 °C, respectively) and longitudinal extension LE (1.05–1.37 mm for the tests conducted at +20 °C and 0.73–0.89 mm for the tests conducted at −20 °C, respectively).

### 5.2. H18N9T Steel

**Figure 10:**
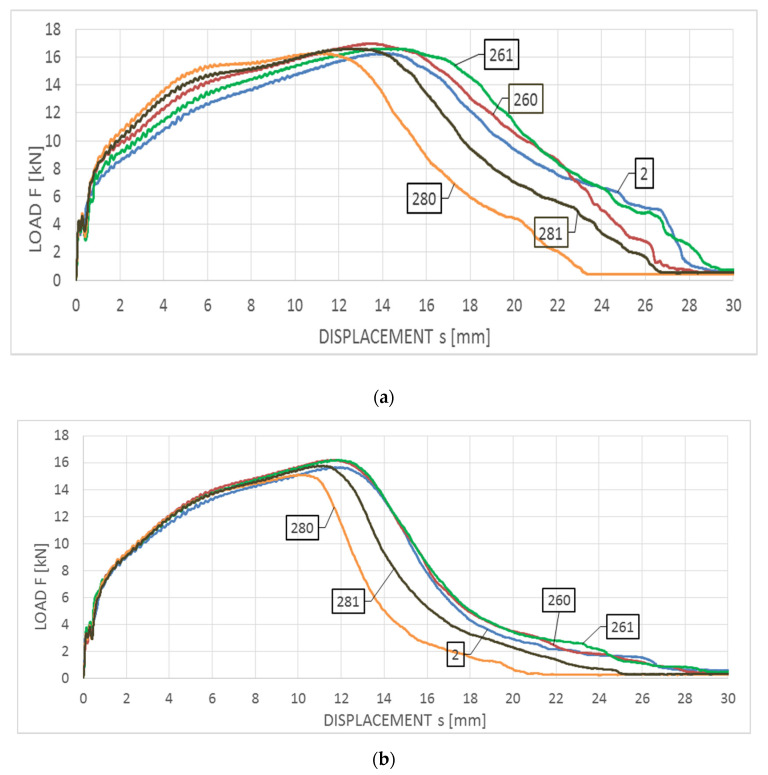
Averaged F−s curves obtained on samples made of 1H18N9T steel: (**a**) during tests conducted in +20 °C, (**b**) during tests conducted in −20 °C.

**Figure 11:**
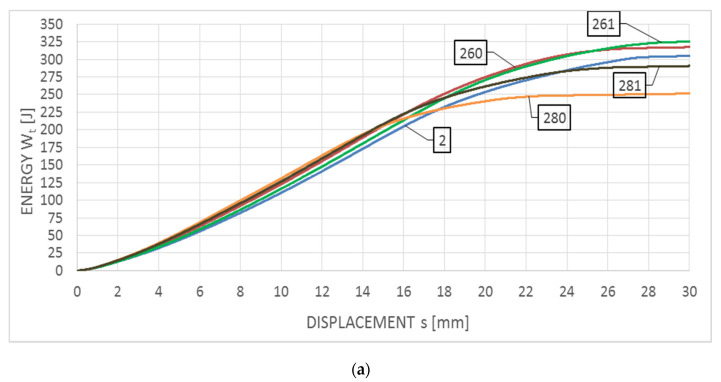

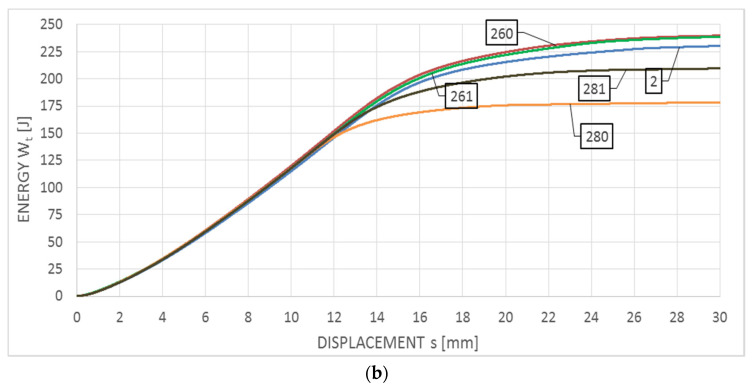
Averaged $W_{t}-s$ curves obtained on samples made of 1H18N9T steel: (**a**) during tests conducted in +20 °C, (**b**) during tests conducted in −20 °C.

Averaged F−s curves obtained for this steel are depicted in Figure 10a with respect to the impact strength tests conducted at the temperature of +20 °C and Figure 10b—with respect to the analogous tests conducted at the temperature of −20 °C. The corresponding $W_{t}-s$ graphs are juxtaposed on Figure 11a,b.

The curves depicted in both figures this time correspond to the F type curves, with plastic zone devoid of unstable crack growth area. All the samples made of this steel, regardless of the testing temperature, are characterized by a fully plastic fracture (though, of course, the steel tested at the temperature of +20 °C proved to exhibit higher plastic properties than the one tested at the temperature of −20 °C). The breaking work $W_{t}$ value obtained during the tests conducted at +20 °C remained within the span of 251–330 J, while the same work obtained during tests conducted at −20 °C remained within the span of 171–240 J. The obtained values of longitudinal extension LE remained within 1.51–1.94 mm (obtained at the temperature of +20 °C) and 1.77–2.00 mm (obtained at the temperature of −20 °C). The largest decrease in the breaking work, with respect to the untreated material, has been observed for this specimen (the group of tests denoted with digit 2) in heating scenarios assuming heating up to 800 °C. This temperature is located in the upper limit of the harmful σ phase precipitation range in the 18/9 class austenitic steels. Slow cooling from this level (the group of tests denoted with number 280) results in a relatively long transfer time of this steel through the zone associated with precipitation of the harmful phase. Fast cooling with water mist (the group of tests denoted with number 281) results in freezing of the microstructure with lower content of the phase σ, and thus lower reduction of the breaking work. Heating of the tested steel at the temperature of 600 °C does not result in the precipitation of this type, thus in such a case reduction of the averaged breaking work, measured in the toughness test of samples subjected to simulated fire action, as compared to the samples made of the material unaffected by simulated fire, did not occur (and even certain increase has been observed), regardless of the temperature at which the tests have been conducted.

### 5.3. X2CrNiMoN22-5-3 Steel

**Figure 12:**
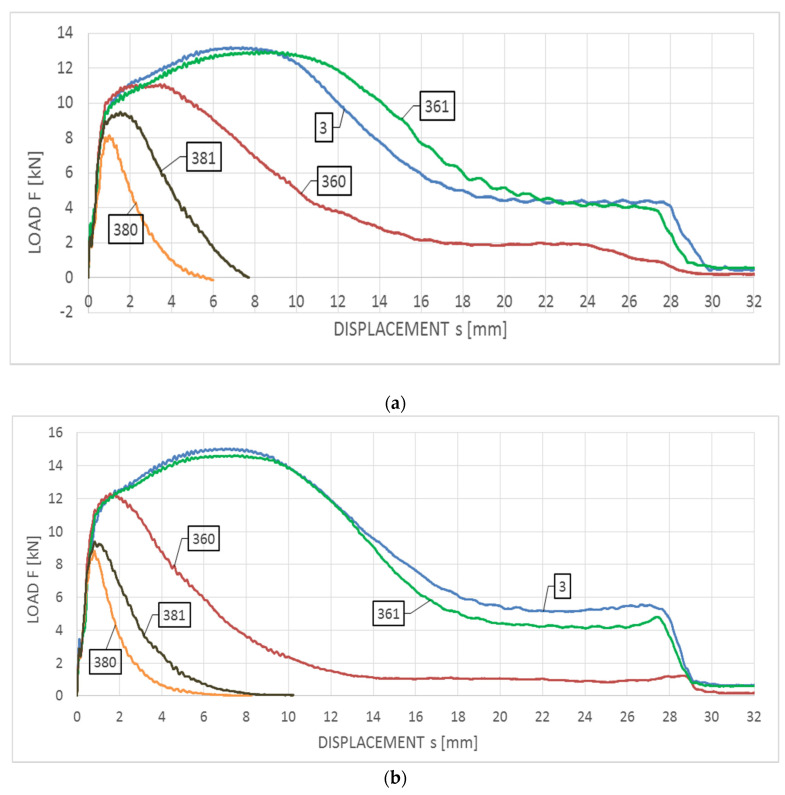
Averaged F−s curves obtained on the samples made of X2CrNiMoN22-5-3 steel: (**a**) during tests conducted in +20 °C, (**b**) during tests conducted in −20 °C.

**Figure 13:**
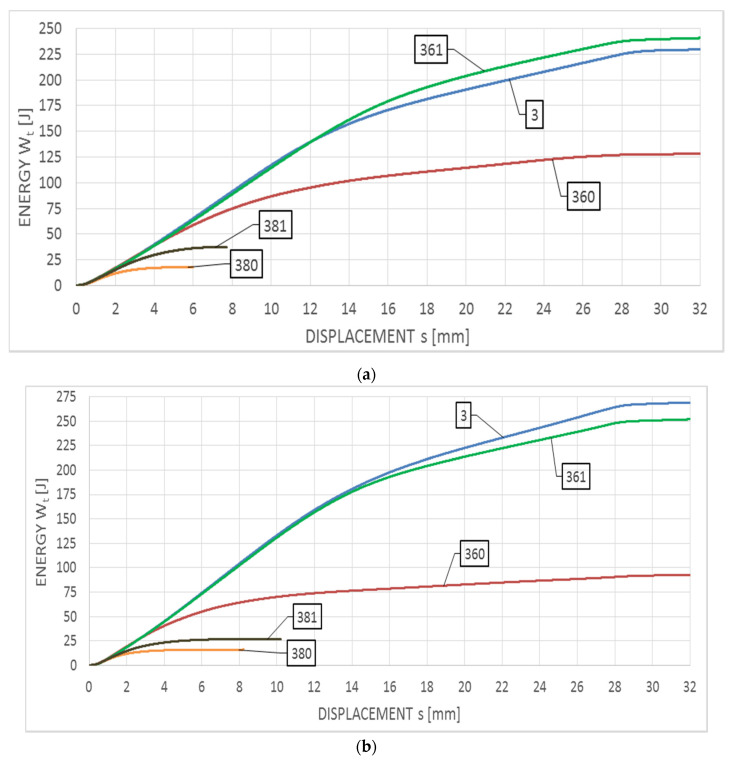
Averaged $W_{t}-s$ curves obtained on samples made of X2CrNiMoN22-5-3 steel: (**a**) during tests conducted in +20 °C, (**b**) during tests conducted in −20 °C.

The averaged impact strength tests authoritative for this steel are juxtaposed in Figure 12a with respect to the tests conducted at +20 °C, and Figure 12b with respect to the tests conducted at −20 °C. The $W_{t}-s$ relationships corresponding to these tests are depicted in Figure 13a,b, respectively.

As may be observed on these graphs, all the curve sets depicted may be assigned to the group F, in spite of substantial differences observed in the breaking work $W_{t}$ obtained for particular scenarios of the simulated fire. All the curves are devoid of unstable crack growth zone. For the samples made of steel remaining in the initial state (a set of samples denoted with digit 3) and samples subjected to the simulated fire restricted to 600 °C during one hour followed by the fast cooling with water (a set of samples denoted with number 361), no significant differences may be observed between the shapes of compared curves depicted in both Figure 13a,b. For this scenario in both cases similar values of breaking work (230 J and 260 J), as well as longitudinal extension (2.10 mm and 2.44 mm), are obtained.

Additionally, the curves characterizing this impact test exhibit a plateau in the final stage of the experiment indicating incomplete break in the specimen. The slow cooling of the sample in the furnace, following the heating to the temperature of 600 °C (a set of samples denoted by the number 360), resulted in slow transition through the critical brittle zone at 475 °C, as a consequence a disadvantageous precipitation of the secondary brittle phases occurred accompanied by a change in the δ ferrite precipitation form to the acicular secondary ferrite α’. This resulted in significant decrease in impact strength, with respect to samples belonging to the group denoted as 361. This simulated fire scenario seems to exhibit the highest sensitivity to the testing temperature for the considered steel, as for the impact notch-toughness test conducted at +20 °C the averaged $W_{t}$ breaking force value at the level of 127 J has been obtained, and averaged longitudinal extension at the level of 2.04 mm. During the tests conducted at −20 °C the respective values of 90 J and 1.44 mm have been obtained.

Fire scenarios, related to the heating temperature of 800 °C during one hour, result in intensive precipitation of numerous carbides, nitrides and intermetallic phases from supersaturated ferrite and austenite. Under such circumstances fast cooling with water (a set of samples denoted with number 381) to a certain degree neutralizes the influence of additional 475 °C brittleness. Unrestricted and slow cooling of the sample in the furnace (a set of samples denoted with number 380) results in successive occurrence of additional harmful brittle phases, thus implicating further decrease in impact strength. In this scenario, in both analyzed cases, low values of the breaking work $W_{t}$ (within the range of 15–35 J) have been obtained.

## 6. Comparative Analysis of the Obtained Fractures

**Figure 14:**
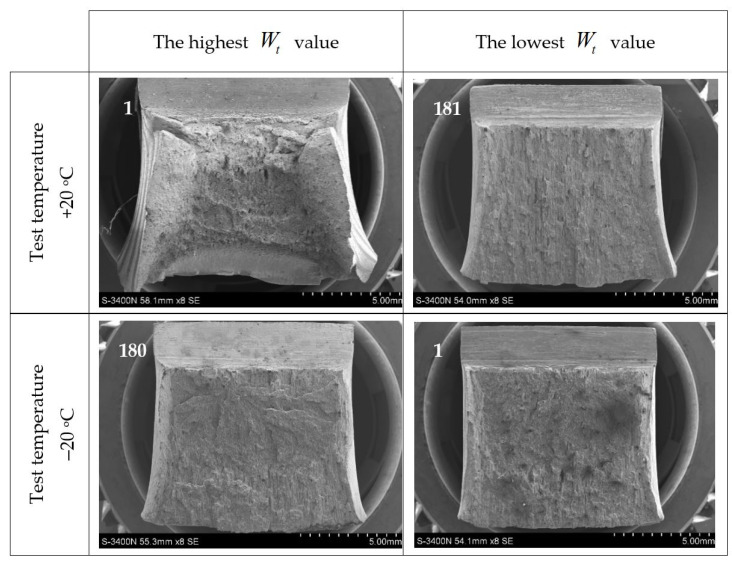
Characteristic impact-notch toughness test fractures obtained on samples made of S355J2+N steel.

**Figure 15:**
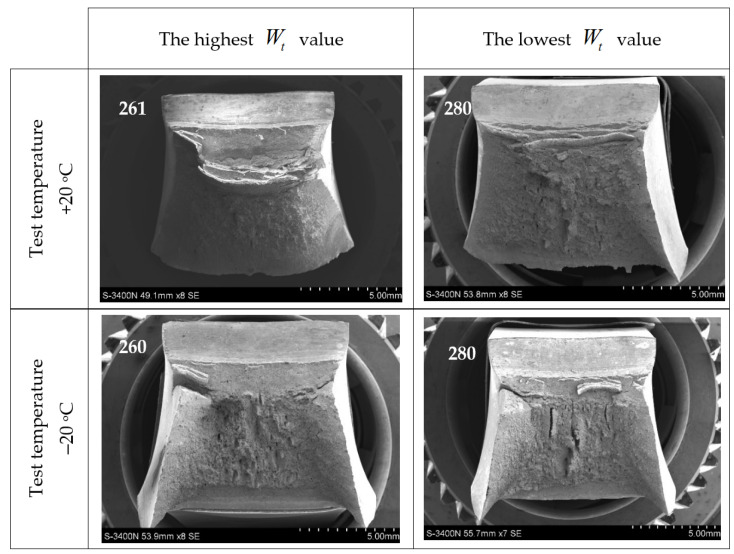
Characteristic impact-notch toughness test fractures obtained on samples made of 1H18N9T steel.

**Figure 16:**
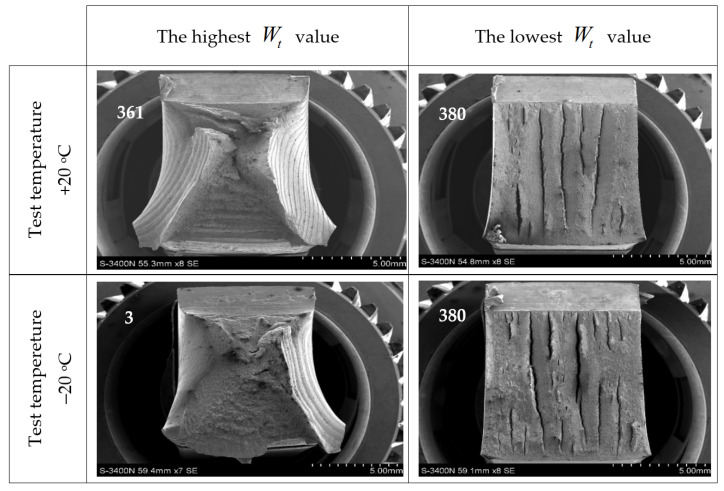
Characteristic impact-notch toughness test fractures obtained on samples made of X2CrNiMoN22-5-3 steel.

The different properties of the steel grades considered in this analysis, cooled down after previous exposure to simulated fire, assessed in the aspect of the verification of forecast capability for plastic deformations in the event that they were admitted for further service, observed in the notch toughness tests presented in this paper (Figure 8 through Figure 13), correspond well with significant differences visible in the fractures obtained. Those fractures are depicted in detail in Figure 14, Figure 15 and Figure 16, where the limit cases with respect to the test temperature (+20 °C and −20 °C) and experimentally determined breaking energy $W_{t}$ of the analysed samples are presented. The white numbers visible in the upper left corner of each photograph identify the sample related to the fracture depicted (according to the system shown in Table 1). 

The characteristic fractures obtained during impact-notch toughness tests performed on S355J2+N steel are depicted in Figure 14. It may be stated, that in the case of sample denoted as 1, at the test temperature of +20 °C, a fully plastic fracture occurred. An intercrystalline, pitted fracture has been observed. A large area of near surface plastic deformations both under the notch bottom and at shear lips has been identified. The sample 181, tested at the temperature of +20 °C is characterized by plastic fracture as well, but this time the degree of development of the cross sectional area of this fracture turned out to be relatively small. The sample 180, tested at the temperature of −20 °C, exhibited a large area of unstable cracking, of partially transcrystalline character under notch bottom. This type of fracture seems to confirm the conclusions drawn by the Authors, preliminarily formulated after the analysis of curves depicted above in Figure 8b. The shear lip zone is visible in this case as well. Therefore it is not surprising, that the unstable crack zone has been observed on the fracture related to the sample 1, but this time tested at the temperature of −20 °C. However, large breakouts of entire crystalline assemblies have been observed here (in the Authors’ opinion this is a consequence of polluted intergranular boundaries). The shear lip extent is visible on fracture of this test sample as well. 

The fractures juxtaposed in Figure 15 pertain to the samples made of 1H18N9T steel. Completely ductile intercrystalline fractures are visible in all pictures. Very strong plastic deformations under notch bottom are observed in the fracture of the sample denoted as 261. It seems to be interesting, that the sample 280, after testing at the temperature of −20 °C exhibited a low tendency to cracking with lamellar transverse delaminations.

Juxtaposition depicted in Figure 16 shows an illustration of impact-notch toughness test fractures obtained on samples of X2CrNiMoN22-5-3 steel. The fractures obtained on samples denoted as 361 and 3 were fully intercrystalline. This is in agreement with results depicted in Figure 12a,b above. A strong deformation observed under notch bottom is characteristic of both these cases. The samples denoted as 380, when tested both at +20 °C as well as at −20 °C exhibited a brittle fracture, with a strong tendency to create transverse lamellar delaminations. This type of fracture, according to Authors’ opinion, corresponds well with the particular shape of the related F−s curves depicted in Figure 12. The small distortions at the pendulum impact point are a consequence of the thinned down cross section of the ISO Charpy V-7.5 sample. 

**Figure 17:**
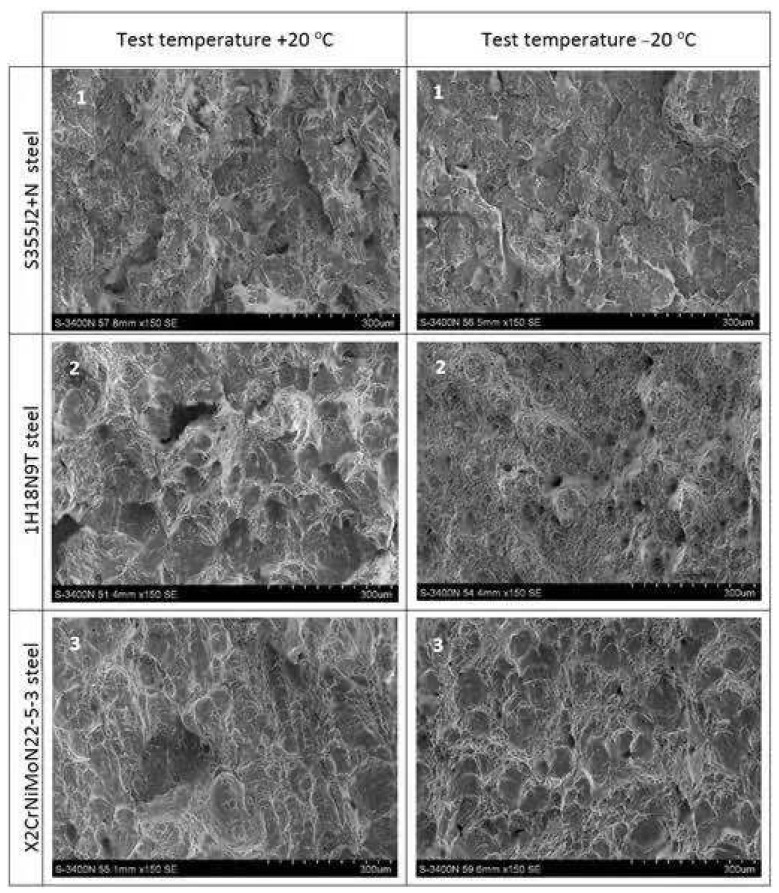
Typical fracture surfaces obtained on samples in the initial state (prior to the action of simulated fire).

**Figure 18:**
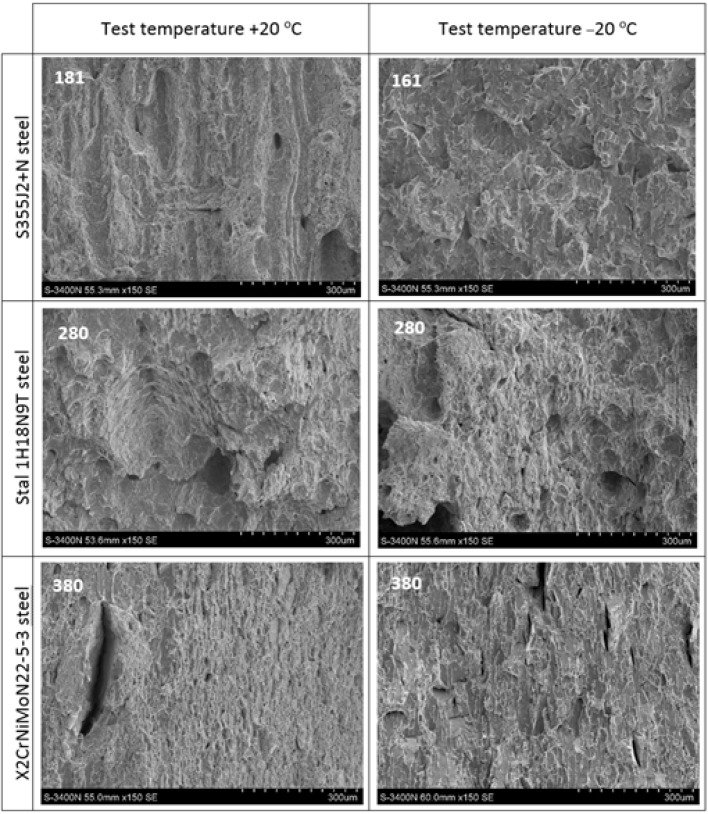
Selected fracture surfaces obtained on samples cooled to room temperature after prior action of fire temperature (description in the text).

In further analysis it will be shown in more detail, at higher magnifications, how the episodes of heating at constant temperature and cooling of the samples made of steels subjected to experiment affected the nature of fractures obtained in the course of the tests conducted. Thus the fracture surfaces typical for each of the considered steel grades remaining in the initial state, i.e., not subjected to the simulated fire action, juxtaposed in the Figure 17, will be compared against fracture surfaces observed on samples cooled after simulated fire action, subjected to several heating and cooling scenarios considered in the experiment. Those fracture surfaces are depicted in the Figure 18.

As may be observed in Figure 17, the samples made of S355J2+N steel remaining in the initial state (denoted with digit 1 at the upper left corner) exhibit fracture surface of the ductile type. The change in testing temperature from +20 °C to −20 °C resulted in significant increase of the transcrystalline fracture at the expense of plastic—pitting fracture. Fracture of the sample made of this steel heated to 800 °C and subsequently cooled in the water mist, denoted in Figure 18 by the number 181 and tested at the temperature of +20 °C exhibited properties of the ductile transcrystalline fracture. In this fire scenario precipitation of the secondary phases occurred at the grain boundaries and as a result the magnitude of the $W_{t}$ energy decreased. However, the precipitates have not been observed on the sample denoted in Figure 18 by the number 161, heated to the temperature of 600 °C only. Due to the lower testing temperature of −20 °C, the fracture obtained exhibited ductile character with dominant transcrystalline component, therefore it proved to be analogous to the fracture of the material remaining in the initial (virgin) state, described above.

On the samples made of 1H18N9T and X2CrNiMoN22-5-3 steels remaining in the virgin state, denoted in Figure 17 with digits 2 and 3, respectively, a plastic pitting fracture has been observed. It has been also observed that with testing temperature lowered from +20 °C to −20 °C the degree of surface development of the obtained fracture decreased significantly, but its character did not change. 

The analysis of samples made of 1H18N9T steel, heated to 800 °C and subsequently slowly cooled in the furnace, denoted in Figure 18 with number 280, allows for the conclusion that in such case the pitting fracture observed on samples made of this material remaining in virgin state, devoid of initial heat treatment, in general did not undergo any important change. However, now the morphology of the described fracture surface additionally indicates the appearance of small subgrain secondary precipitates, responsible for the reduction of $W_{t}$ energy. Furthermore, when testing both at +20 °C as well as at −20 °C, small transverse lamellar delaminations of ductile transcrystalline character appear on the fracture surfaces depicted in Figure 18 (denoted with symbol 280).

The samples made of X2CrNiMoN22-5-3 steel heated to the temperature of 800 °C and after subsequent cooling tested at +20 °C, therefore denoted in Figure 18 by number 380, exhibit a brittle intercrystalline fracture, with small subgrain secondary precipitates appearing at grain boundaries. The samples tested at −20 °C exhibit a brittle transcrystalline fracture surface. In both cases considered the surfaces of transverse lamellar delaminations exhibit brittle transcrystalline character.

## 7. Concluding Remarks

The results obtained in the impact notch-toughness tests conducted, and related to selected conditions simulating prior action of fire temperature on the analysed samples followed by two sample cooling scenarios after fire seem to indicate different behaviour of each tested steel grade when subjected to such conditions. Thus they may constitute a basis for drawing relatively reliable conclusions regarding post-fire susceptibility of a given material to the initiation and propagation of brittle cracks, and this seems to be crucial for the assessment of its suitability for further use. Of the steels covered in this research, especially the steels similar in behaviour to the S355J2+N grade seem to be remarkably prone to the destruction of this type. This statement refers in particular to the winter conditions, as under those conditions a zone of unstable crack growth manifests itself in a pronounced manner. Let us note, however, that the simulated fire scenario, which, of all considered, proved itself to affect the impact strength of steel S355J2+N in a least adverse manner, at the same time affected the impact strength of steel 1H18N9T in the most adverse manner. The X2CrNiMoN22-5-3 steel exhibited during tests very high sensitivity to the heating temperature. Its susceptibility to brittle fracture after heating to 800 °C during one hour proved to be substantially higher than the one obtained after heating to only 600 °C during the same time. At the lower heating temperature the cooling regimen substantially affects the final impact strength, as the slow cooling in the furnace results in substantial decrease in breaking work measured in experiment, especially in the simulated winter conditions.

Reliability of the above conclusions, formulated based on the detailed analysis of experimentally determined F−s curves seems to be unequivocally confirmed in the qualitative as well as quantitative terms by an independent fractographic analysis of fracture surfaces observed on individual samples.

## Figures and Tables

**Table 1:** Description mode of the samples subjected to impact testing.

First Digit—Steel Grade	Second Digit—Heating Temperature	Third Digit—Cooling Mode	Test Temperature
1—S355 2—1H18N9T 3—X2CrNiMoN22-5-3	6—600 °C 8—800 °C	0—cooling in the furnace 1—cooling in water mist	+20 °C −20 °C

## Data Availability

The data presented in this study are available on request from the corresponding author.
